# WSL5, a pentatricopeptide repeat protein, is essential for chloroplast biogenesis in rice under cold stress

**DOI:** 10.1093/jxb/ery214

**Published:** 2018-06-08

**Authors:** Xi Liu, Jie Lan, Yunshuai Huang, Penghui Cao, Chunlei Zhou, Yaken Ren, Niqing He, Shijia Liu, Yunlu Tian, Thanhliem Nguyen, Ling Jiang, Jianmin Wan

**Affiliations:** 1 State Key Laboratory for Crop Genetics and Germplasm Enhancement, Jiangsu Plant Gene Engineering Research Center, Nanjing Agricultural University, Nanjing, China; 2 National Key Facility for Crop Gene Resources and Genetic Improvement, Institute of Crop Science, Chinese Academy of Agricultural Sciences, Beijing, China

**Keywords:** Chloroplast development, cold stress, *Oryza sativa*, ribosome biogenesis, RNA-seq, RNA splicing

## Abstract

Chloroplasts play an essential role in plant growth and development, and cold conditions affect chloroplast development. Although many genes or regulators involved in chloroplast biogenesis and development have been isolated and characterized, many other components affecting chloroplast biogenesis under cold conditions have not been characterized. Here, we report the functional characterization of a *white stripe leaf 5* (*wsl5*) mutant in rice. The mutant develops white-striped leaves during early leaf development and is albinic when planted under cold stress. Genetic and molecular analysis revealed that *WSL5* encodes a novel chloroplast-targeted pentatricopeptide repeat protein. RNA sequencing analysis showed that expression of nuclear-encoded photosynthetic genes in the mutant was significantly repressed, and expression of many chloroplast-encoded genes was also significantly changed. Notably, the *wsl5* mutation causes defects in editing of *rpl2* and *atpA*, and splicing of *rpl2* and *rps12*. *wsl5* was impaired in chloroplast ribosome biogenesis under cold stress. We propose that the *WSL5* allele is required for normal chloroplast development in maintaining retrograde signaling from plastids to the nucleus under cold stress.

## Teaser

*WSL5* is crucial for the splicing of the chloroplast genes *rpl2* and *rps12* under cold stress, and affects the retrograde signaling from plastids to the nucleus.

## Introduction

Cold is an important environmental factor affecting chloroplast development and growth in juvenile plants. Sudden low temperature events that often occur during early seedling development in spring can directly affect chlorophyll development (Kusumi and Iba, 2014). Rice seedlings are susceptible to cold stress, with an impact that ultimately affects grain yield (Liu *et al.*, 2013). Therefore, cold stress is a common problem that affects grain production, and rice varieties with increased cold tolerance are preferred (Zhao *et al.*, 2017). Many studies have suggested that plants can regulate early chloroplast development under cold stress. The chlorophyll content in young leaves of a virescent mutant was low, but gradually increased to normal levels as they grew (Yoo *et al.*, 2009). Temperature-sensitive virescent mutants were used to study mechanisms regulating chloroplast development in seedlings under cold stress, and many genes were identified (e.g. *V3*, *St1*, *OsV4*, *TCD9*, and *TSV*) (Yoo *et al.*, 2009; Gong *et al.*, 2014; Jiang *et al.*, 2014; Sun *et al.*, 2017). However, the mechanisms of chloroplast development in rice seedlings under cold stress remain poorly understood.

Chloroplasts are essential for photosynthesis and have crucial roles in plant development and growth by fixation of CO_2_ and biosynthesis of carbon skeletons, as well as other physiological processes (Jarvis and López-Juez, 2013). Formation of a photosynthetically active chloroplast from a proplastid is controlled by both nuclear-encoded polymerase (NEP) and plastid-encoded polymerase (PEP) genes (Yu *et al.*, 2014). NEP is a single protein that is responsible for the transcription of genes encoding PEP subunits, ribosomal proteins, and other plastidic ‘housekeeping’ proteins (Liere *et al.*, 2011). PEP, on the other hand, is a large, dynamic complex with many transiently attached peripheral subunits (Yu *et al.*, 2014).

Chloroplast RNAs need to be processed to become functional rRNAs and mRNAs. Many of the processing factors for RNA cleavage, splicing, editing, and stability are RNA-binding proteins (Tillich and Krause, 2010). All are encoded by the nuclear genome. Pentatricopeptide repeat (PPR) proteins are a family of RNA-binding proteins that usually carry out specific RNA processing in chloroplasts (Stern *et al.*, 2010; Shikanai and Fujii, 2013). PPR proteins are defined by tandem arrays of a degenerate 35 amino acid repeat (PPR motif). In higher plants, the PPR family comprises many members, with 450 in Arabidopsis and 655 in rice (O’Toole *et al.*, 2008). The functions of PPR proteins are well characterized (Stern *et al.*, 2010; Shikanai and Fujii, 2013). Chloroplast-targeted PPR proteins were characterized as being involved in regulating RNA splicing, RNA editing, RNA stability, and RNA translation during plant development and growth (Yu *et al.*, 2009; Ichinose *et al.*, 2012). Several PPR genes in rice, such as *YSA*, *OsV4*, *WSL*, *ALS3*, *OspTAC2*, and *WSL4*, were reported to function in RNA editing, RNA splicing and chloroplast development (Su *et al.*, 2012; Gong *et al.*, 2014; Tan *et al.*, 2014; Lin *et al.*, 2015; D. Wang *et al.*, 2016; Wang *et al.*, 2017). The rice PPR mutant *ysa* develops albinic leaves before the three-leaf stage, but the plants gradually turn green and recover to normal green at the six-leaf stage (Su *et al.*, 2012). *WSL* encodes a PPR protein that is targeted to the chloroplast and plays an essential role in the splicing of *rpl2* (Tan *et al.*, 2014). The P-family PPR mutant *wsl4*, which exhibits white-striped leaves before the five-leaf stage, has defective chloroplast RNA group II intron splicing (Wang *et al.*, 2017). However, the functions, substrates, and regulatory mechanisms of many PPR proteins in rice remain to be elucidated.

In this study, we isolated and characterized the rice mutant *wsl5* that develops white-striped leaves at the early seedling stage. *wsl5* is albinic under low temperatures. *WSL5* encodes a P-family PPR protein containing an RNA recognition motif (RRM) at its N-terminus and 15 PPR motifs at its C-terminus. WSL5 locates to the chloroplast and is essential for chloroplast ribosome biogenesis under cold stress. We showed that RNA editing sites of *rpl2* and *atpA* were not edited, and plastid-encoded genes *rpl2* and *rps12* were not efficiently spliced in the *wsl5* mutant. Abnormal splicing of *rpl2* and *rps12* may lead to chloroplast failure to form functional ribosomes, which blocks retrograde signaling from chloroplasts to the nucleus. Our results provide novel insights into the function of *WSL5* in regulating rice chloroplast development under cold stress.

## Materials and methods

### Plant materials and growth conditions

The *wsl5* mutant was isolated from an ethylmethane sulfonate (EMS) mutant pool of *indica* cultivar Nanjing 11. Seedlings were grown in a growth chamber under 16 h of light/8 h of darkness at constant temperatures of 20, 25, and 30 °C. The third leaves at ~10 d post-planting were used for nearly all analyses. To map the *WSL5* locus, we constructed an F_2_ population derived from a cross of the *wsl5* mutant and Dongjin (*japonica*).

### Pigment determination and TEM

Wild-type and *wsl5* mutant seedlings were grown in the field. Fresh leaves were collected and used to determine chlorophyll contents using a spectrophotometer according to the method of Arnon (1949). Briefly, 0.2 g of leaf tissue was collected, marinated in 5 ml of 95% ethanol, and held for 48 h in darkness. The supernatants were collected by centrifugation and were analyzed with a DU 800 UV/Vis Spectrophotometer (Beckman Coulter) at 665, 649, and 470 nm, respectively.

TEM was performed according to the method of Y. Wang *et al.* (2016). Briefly, fresh leaves were collected and cut into small pieces, fixed in 2.5% glutaraldehyde in a phosphate buffer at 4 °C for 4 h, further fixed in 1% OsO_4_, stained with uranyl acetate, dehydrated in an ethanol series, and finally embedded in Spurr’s medium prior to ultrathin sectioning. The samples were observed using a Hitachi H-7650 transmission electron microscope.

### Map-based cloning and complementation of WSL5

Genetic analysis was performed using an F_2_ population (*wsl5*/Nanjing 11); 654 plants with the recessive mutant phenotype were used for genetic mapping. New simple sequence repeat (SSR)/Indel markers were developed based on the Nipponbare (*japonica*) and 93-11 (*indica*) genome sequences (http://www.gramene.org/). The *WSL5* locus was narrowed to a 180 kb region between markers Y18 and Y47 on the long arm of chromosome 4 (see Table S2 available at the Dryad Digital Repository, http://dx.doi.org/10.5061/dryad.59185).

For complementation of the *wsl5* mutation, a 2706 bp wild-type coding sequence fragment and an ~2 kb upstream sequence were amplified from variety Nanjing 11. They were cloned into the binary vector pCAMBIA1390 to generate the vector pCAMBIA1390-*WSL5*. This vector was introduced into *Agrobacterium tumefaciens* strain EHA105, which was then used to infect *wsl5* mutant calli.

### Sequence analysis

Gene prediction and structure analysis were performed using the GRAMENE database (www.gramene.org/). Homologous sequences of WSL5 were identified using the Blastp search program of the National Center for Biotechnology Information (NCBI, www.ncbi.nlm.nih.gov/). Multiple sequence alignments were conducted with DNAMAN.

### Subcellular localization of WSL5 protein

For subcellular localization of WSL5 protein in rice, the coding sequence of *WSL5* was amplified and inserted into the pAN580 vector. The cDNA fragments were PCR-amplified using primer pairs shown in Table S2 at Dryad. Transient expression constructs were separately transformed into rice protoplasts and incubated in darkness at 28 °C for 16 h before examination (Chen *et al.*, 2006). Green fluorescent protein (GFP) fluorescence was observed using a confocal laser scanning microscope (Zeiss LSM 780).

### Quantitative RT-PCR (qRT-PCR) analysis

Total RNA was isolated using the RNA prep pure plant kit (TIANGEN, Beijing). First-strand cDNA was synthesized using random hexamer primers (TaKaRa) for chloroplast-encoded genes and oligo(dT)_18_ (TaKaRa) for nuclear-encoded genes, and reverse transcribed using Prime scriptase (TaKaRa). RT-PCR was performed in three biological repeats using an ABI 7500 real-time PCR system with SYBR Green Mix. Primers used for RT-PCR are listed in Table S2 at Dryad. The rice *Ubiquitin* gene was used as an internal control.

### RNA analysis

Total RNA was isolated from 10-day-old seedlings of the wild type and *wsl5* grown in C30 and C20 conditions using an RNA prep pure plant kit. RNA samples were diluted to 10 ng m^–1^ and analyzed by an Agilent 2100 bioanalyzer. The RNA 6000 Nano Total RNA Analysis Kit (Agilent) was used for analysis.

### RNA editing sites and RNA splicing analysis

Specific cDNA fragments were generated by RT-PCR amplification following established protocols (Takenaka and Brennicke, 2007). The cDNA sequences were compared to identify C to T changes resulting from RNA editing. For RNA splicing analysis, chloroplast genes with at least one intron were selected and amplified using RT-PCR with primers flanking the introns. The primers used for RNA editing and splicing analysis were obtained as reported previously (Tan *et al.*, 2014; Zhang *et al.*, 2017).

### Protein extraction, SDS–PAGE, and western blotting

Leaf material was homogenized in lysis buffer (25 mM Tris–HCl, pH 7.6, 0.15 M NaCl, 2% SDS, 0.01% 2-mercaptoethanol). Sample amounts were standardized by fresh weight. The protein samples were separated by 10% SDS–PAGE. After electrophoresis, the proteins were transferred onto polyvinylidene difluoride (PVDF) membranes (Millipore) and incubated with specific antibodies. Signals were detected using an ECL Plus Western Blotting Detection Kit (Thermo) and visualized by an imaging system (ChemiDocTMX- RS; Bio-Rad).

### Yeast two-hybrid analysis

The coding sequences of five rice multiple organellar RNA editing factors (MORFs) were amplified with primers listed in Zhang *et al.* (2017). MORFs and WSL5 were cloned into the pGAD-T7 or pGBK-T7 vectors, respectively. Yeast two-hybrid analysis was performed using the Clontech (www.clontech.com) two-hybrid system, following the manufacturer’s instructions.

### RNA sequencing (RNA-seq) analysis

Total RNA was extracted from leaves of 10-day-old wild-type and *wsl5* seedlings grown at different temperatures. mRNA was enriched from total RNA using oligo(dT) primers and Ribo-Zero rRNA Removal Kits for chloroplast-encoded genes. cDNA was synthesized using random hexamer primers. The library was constructed and sequenced using an Illumina Hisequation 2000 (TGS, Shenzhen). Totals of 45 million reads of genes from the wild type and 42 million from *wsl5* were obtained. The significance of differentially expressed genes (DEGs) was determined by using |log_2_ (fold change)|>1 and *q*-values <0.05. Gene Ontology (http://www.geneontology.org/) analyses were performed referring to GOseq (Young *et al.*, 2010). Pathway enrichment analysis was performed using the Kyoto Encyclopedia of Genes and Genomes (KEGG) database (Kanehisa *et al.*, 2008).

## Results

### Characterization of the wsl5 mutant

**Fig. 1.**
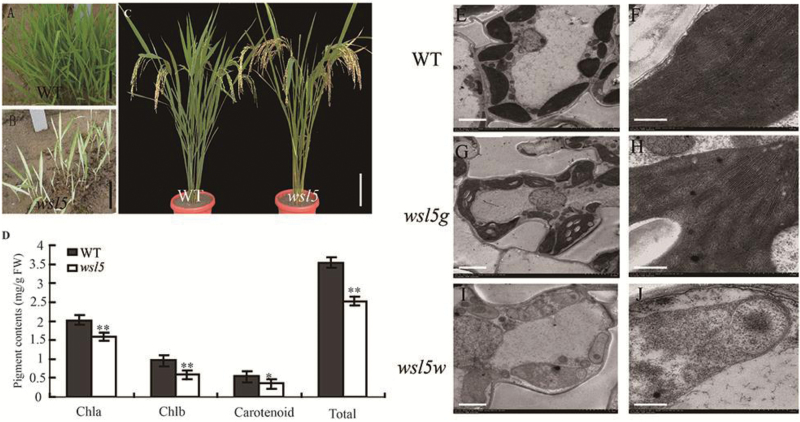
Phenotypic characteristics of the *wsl5* mutant. (A, B) Phenotypes of wild-type (WT) and *wsl5* mutant seedlings in the field 20 d after seeding. (C) Phenotypes of WT (left) and *wsl5* (right) plants at maturity. (D) Leaf pigment contents of field-grown WT and *wsl5* seedlings at 20 d after seeding. (E, F) Mesophyll cells in WT plants showing normal, well-ordered chloroplasts. (G, H) Chloroplasts from green sectors of *wsl5* seedlings were indistinguishable from those of the WT. (I, J) Cells from white sectors of the mutants displayed abnormalities, including vacuolated plastids and lack of organized thylakoid membranes. Scale bar=1 cm in (A, B), 10 cm in (C), 1 μm in (E, G, I), 500 nm in (F, H, J) (Student’s *t*-test, **P<0.01, *P<0.05).

To identify genetic factors regulating chloroplast development in rice, we used the *wsl5* mutant obtained in an EMS mutant pool of Nanjing 11 (*indica*). Seedlings of *wsl5* exhibited a white-striped leaf phenotype up to the four-leaf stage under field conditions (Fig. 1A, B). Normal green leaves occurred thereafter. Chlorophyll (Chl *a* and Chl *b*) and carotenoid contents were reduced in the *wsl5* mutant seedlings before the five-leaf stage, but were subsequently similar to those of the wild type (Fig. 1D; Fig. S1 at Dryad). The major agronomic traits of the *wsl5* mutant at maturity, including plant height and grain size, were indistinguishable from those of wild-type plants (Fig. 1C; Table S1 at Dryad). To examine whether the color deficiency was accompanied by ultrastructural changes in chloroplasts, we compared the ultrastructure of chloroplasts in white and green sectors of *wsl5* mutant leaves and normal wild-type leaves by TEM. Cells in wild-type leaves and green sectors in leaves of *wsl5* had normal chloroplasts displaying structured thylakoid membranes composed of grana connected by stroma lamellae (Fig. 1E, H). However, the white sectors of *wsl5* had abnormal chloroplasts (Fig. 1I, J). The results suggested that *WSL5* had a role in chloroplast development in juvenile plants.

### The *wsl5* phenotype was temperature sensitive

**Fig. 2.**
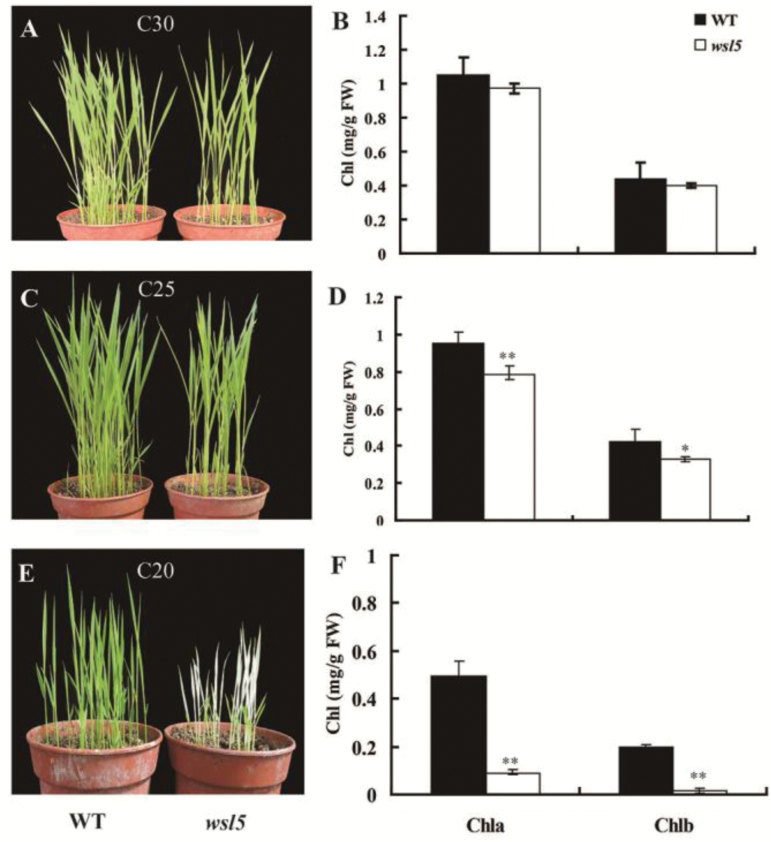
The *wsl5* phenotype is temperature sensitive. (A, C, E) Phenotypes of wild-type (WT) and *wsl5* seedlings produced at different constant temperatures. C20, C25, and C30, refer to 20, 25, and 30 °C, respectively. (B, D, F) Leaf pigment contents of WT and *wsl5* seedlings grown at different temperatures (Student’s *t*-test, *P<0.05, **P<0.01).

To verify whether the *wsl5* mutant was affected by temperature, *wsl5* and wild-type seedlings were grown in growth chambers under constant temperatures of 20, 25, and 30 °C (C20, C25, and C30). Leaves of the *wsl5* mutant were albinic at 20 °C (Fig. 2E) and the plants died. Chlorophyll was not detectable in the leaves (Fig. 2F). At 25 °C, the *wsl5* mutant developed leaves with white stripes and reduced chlorophyll content (Fig. 2C, D). At 30 °C, *wsl5* exhibited almost the same phenotype as the wild type (Fig. 2A) and contained similar pigment contents (Fig. 2B). These results suggested that the lack of WSL5 protein at 20 °C resulted in the observed damage.

We also examined the ultrastructure of chloroplasts in mesophyll cells of wild-type and *wsl5* plants. At 30 °C, all the wild-type and *wsl5* plants displayed normal chloroplasts with well-developed lamellar structures and normally stacked grana and thylakoid membranes (Fig. S2A–D at Dryad). At 20 °C, the wild type developed large starch grains and chloroplasts with normal thylakoids (Fig. S2E, F at Dryad), whereas leaf cells from albinic sectors in *wsl5* had no chloroplasts (Fig. S2G, H at Dryad). The results indicated that the lack of WSL5 protein at 20 °C resulted in the observed damage.

### Map-based cloning of the *WSL5* allele

**Fig. 3.**
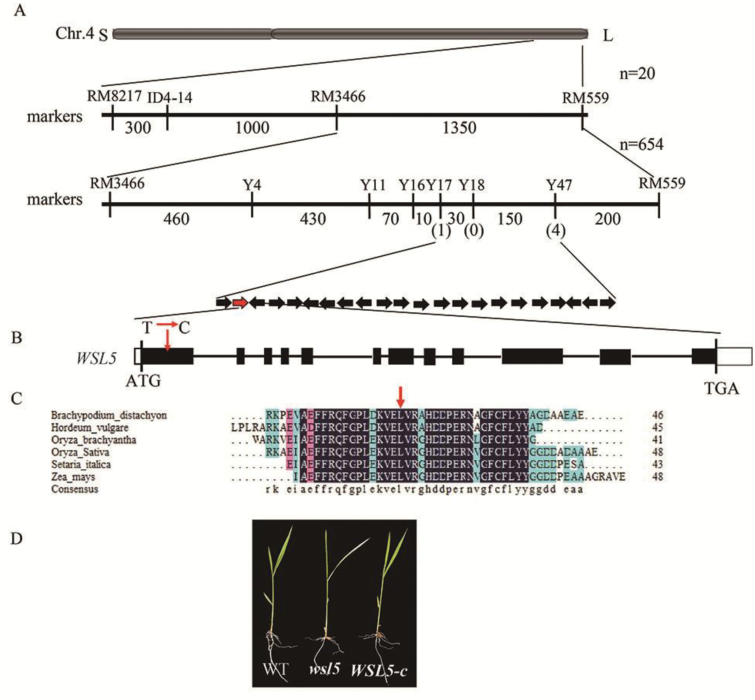
Map-based cloning of the *WSL5* allele. (A) The *WSL5* locus was mapped to a 180 kb region between markers Y17 and Y47 on chromosome 4L. Black arrows represent 22 putative genes in this region; the candidate gene *WSL5* (*Os04g0684500*) is shown by a red arrow. (B) ATG and TGA represent the start and stop codons, respectively. Black boxes indicate the exons, and the white boxes indicate the 3'- and 5'-UTRs. An SNP in the first exon in *WSL5* causes a leucine to proline amino acid substitution. (C) Alignment of amino acid sequences with highest identity to the WSL5 protein. The red arrow indicates an amino acid change. (D) Complementation of *wsl5* by transformation.

Genetic analysis showed that the white stripe phenotype of the *wsl5* mutant was controlled by a single recessive nuclear locus. To identify the *WSL5* locus, 20 F_2_ individuals with the mutant phenotype derived from a cross between *wsl5* and Dongjin (*japonica*) were used. The *WSL5* locus was located to a 2.65 Mb region between markers RM8217 and RM559 on the long arm of chromosome 4. It was further delimited to a 180 kb region between markers Y17 and Y47 using 654 F_2_ plants with the mutant phenotype. Twenty-two ORFs were predicted in the region from published data (http://www.gramene.org/;Fig. 3A). Sequence analysis of the region showed that only one ORF encoding a PPR protein differed between the wild type and *wsl5* (Fig. 3B). A single nucleotide polymorphism (SNP; T to C) located in the conserved region caused a leucine to proline amino acid substitution in the mutant (Fig. 3B, C).

To confirm that mutation of *WSL5* was responsible for the mutant phenotype, the *WSL5* coding region driven by the *UBQ* promoter was transformed into calli derived from *wsl5*. Twenty-eight of 45 transgenic lines resistant to hygromycin and harboring the transgene displayed the wild-type phenotype (Fig. 3D). These results confirmed that *Os04g0684500* was *WSL5*.

### *WSL5* encodes a PPR protein

Sequence analysis showed that WSL5 comprised 12 exons and 11 introns. The single base substitution in *wsl5* was located in the first exon (Fig. 3B). A database search with Pfam (http://pfam.xfam.org/search) revealed that WSL5, which belongs to the P family, contained an RRM at its N-terminus and 15 PPR motifs at the C-terminus. The substituted amino acid (leucine) was highly conserved in the RRM of homologous proteins (Fig. 3C), suggesting an obligate role for this site in functional integrity of WSL5 protein. WSL5 shared a high degree of sequence similarity with maize PPR4 (84% identity) and *Arabidopsis thaliana* At5g04810 (59% identity) (Fig. S3 at Dryad). Together, these results indicated that *WSL5* encodes a novel PPR protein.

### Expression pattern and subcellular localization of WSL5

**Fig. 4.**
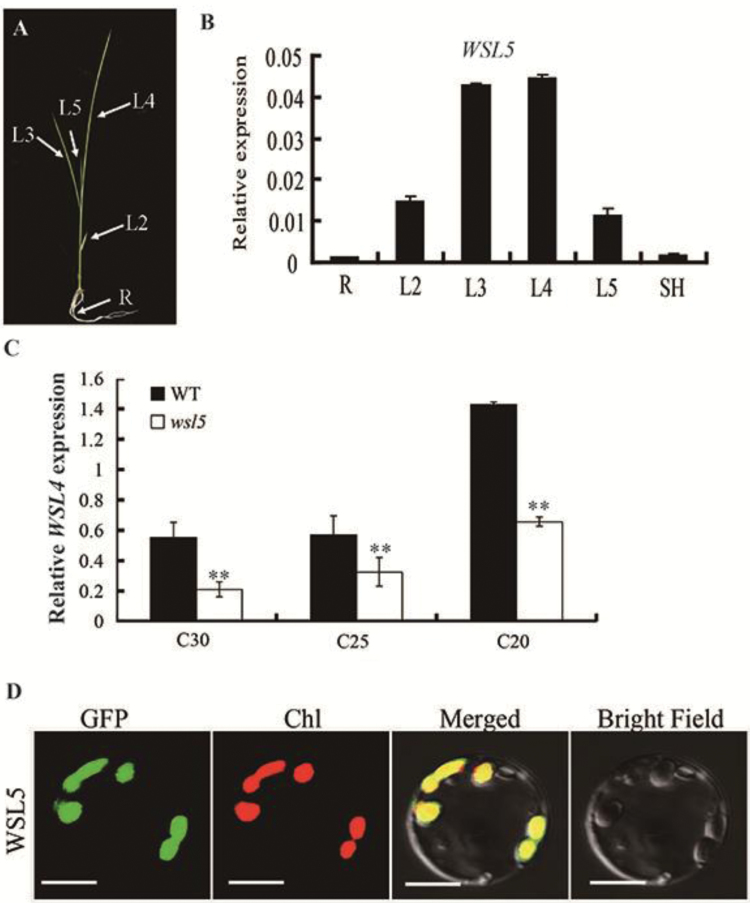
Expression pattern analysis and subcellular localization of WSL5. (A) Schematic of a rice seedling with a fully expanded fourth leaf. (B) qRT-PCR analysis of *WSL5* expression in roots, stems, L2, L3, L4, L5, and sheaths of the wild type (WT). (C) qRT-PCR analyses of the *WSL5* transcript in WT and *wsl5* mutant seedlings grown in a growth chamber with a 12 h photoperiod at 30, 25, and 20 °C. (D) Localization of WSL5 protein in rice protoplasts. Green fluorescence shows GFP, red fluorescence shows chloroplast autofluorescence, and yellow indicates the two types of fluorescence merged. Error bars represent the SD from three independent experiments (Student’s *t*-test, **P<0.01).

Using the Rice eFP Browser (http://bar.utoronto.ca/efprice/cgi-bin/efpWeb.cgi), we found that *WSL5* was expressed in all tissues, especially in young leaves. To verify these data, we examined the expression levels of *WSL5* in different organs of the wild type by qRT-PCR (Fig. 4A, B). The *WSL5* transcript was preferentially expressed in young leaves (Fig. 4B), suggesting that *WSL5* had an important role in chloroplast development in young seedlings. The *WSL5* transcript was more abundant in plants grown at 20 °C than at 30 °C, indicating that *WSL5* was induced by low temperature. Thus plants might express *WSL5* abundantly to regulate chloroplast development under cold stress (Fig. 4C).

To examine the actual subcellular localization of WSL5, a *Cauliflower mosaic virus* (CaMV) 35S-driven construct with a WSL5–GFP fusion protein was generated using the pAN580 vector and transiently expressed in rice protoplasts. Green fluorescent signals of WSL5–GFP co-localized with the autofluorescent signals of chlorophyll (Fig. 4D), suggesting that WSL5 localized to chloroplasts. These results, together with chloroplast localization and the observed *wsl5* phenotype, supported the notion that *WSL5* plays an important role in regulating chloroplast development in rice seedlings.

### Expression of photosynthesis-related genes is down-regulated in *wsl5*

**Fig. 5.**
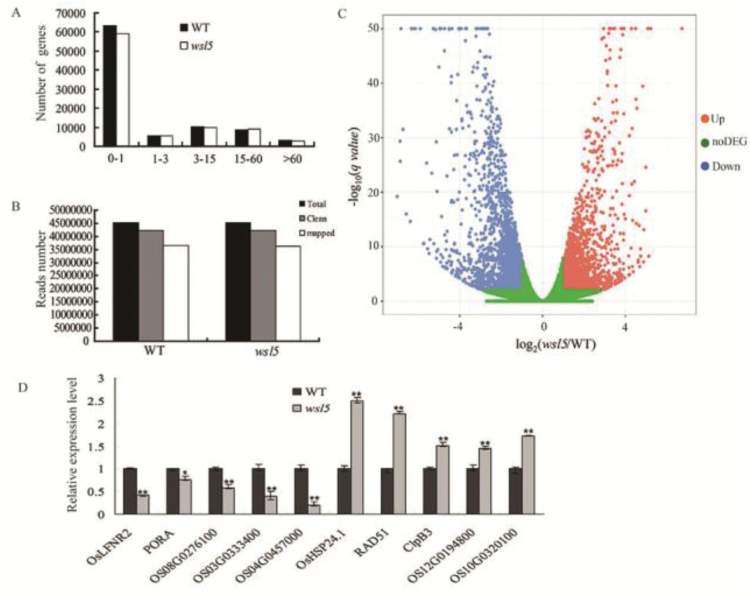
RNA-seq analysis of wild-type (WT) and *wsl5* seedlings grown in C25 conditions. mRNA was enriched using oligo(dT) primers from total RNA isolated from 10-day-old (third leaf) seedlings of the WT and *wsl5* using oligo(dT). cDNA was synthesized using random hexamer primers. The library was then constructed and sequenced using an Illumina HiSEquation 2000. (A) Frequencies of detected genes sorted according to expression levels. (B) Read numbers of WT and *wsl5* sequences. (C) Volcano plot showing the overall alterations in gene expression in the wild type and *wsl5*. (D) qRT-PCR analysis of genes differentially expressed in RNA-seq. Five up-regulated and five down-regulated genes were tested. Error bars represent the SD from three independent experiments (Student’s *t*-test, *P<0.05, **P<0.01).

RNA-seq was performed to analyze the effect of the *wsl5* mutation on gene expression. A total of 42 million clean reads were obtained from the wild type and *wsl5* grown in C25 conditions. Compared with the wild type, there were 1699 up-regulated genes and 1999 down-regulated genes in *wsl5* (Fig. 5A–C; Dataset S1 at Dryad). We randomly selected five down-regulated and five up-regulated genes to verify the results of RNA-seq. The qRT-PCR results were consistent with those from RNA-seq (Fig. 5D). GO and KEGG enrichment analysis indicated that the expression of genes encoding photosynthesis, light reaction, PSI and PSII, chloroplast thylakoid, ATP synthase, and carbon fixation were reduced in *wsl5* (Figs S5, S6 at Dryad). Also, some chlorophyll synthesis genes, including *HEMA*, *YGL8*, *PORA*, *CHLH*, and *CRD1*, were significantly reduced, which was verified using RT-PCR (Fig. S7 at Dryad).

### *wsl5* mutants have global defects in plastidic gene expression

**Fig. 6.**
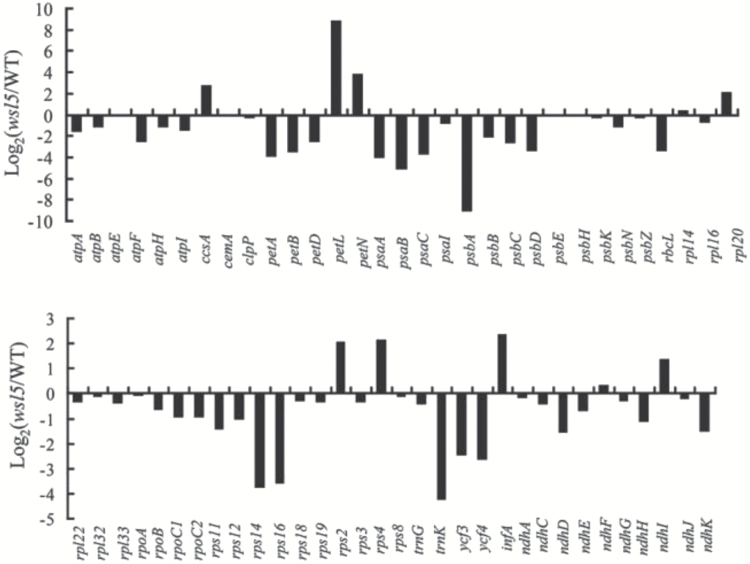
Differential expression of plastid-encoded genes in the wild type (WT) and *wsl5* grown in C25 conditions. mRNA was enriched from total RNA using Ribo-Zero rRNA Removal Kits for chloroplast-encoded genes from 10-day-old seedlings of the WT and *wsl5.* mRNA was fragmented and reverse-transcribed using random hexamer primers. The library was then constructed and sequenced using an Illumina HiSEquation 2000. The graph shows the log_2_ ratio of transcript levels in the *wsl5* mutant compared with the WT.

To investigate whether the *WSL5* mutation affects transcription by PEP and NEP, we examined the transcript abundance of various plastidic genes in the *wsl5* mutant grown in C25 conditions by RNA-seq. The expression of many plastidic genes differed between *wsl5* and the wild type (Fig. 6). Compared with the wild type, the expression of plastidic genes that are transcribed by PEP, including *psbA*, *psbB*, *psbD*, *petB*, *ndhA*, and *rbcL*, was strongly reduced in the *wsl5* mutant. In addition, the transcript levels of plastidic genes, including *ribosomal protein L32* (*rpl32*), *rpl14*, *rps2*, *rps4*, and *rpoA*, which are transcribed by NEP, were increased or unchanged in the mutant (Fig. 6). These results indicated that the *wsl5* mutation influenced the optimal expression of plastidic genes in rice seedlings.

### Analysis of transcripts and proteins of genes associated with chloroplast biogenesis in *wsl5*

**Fig. 7.**
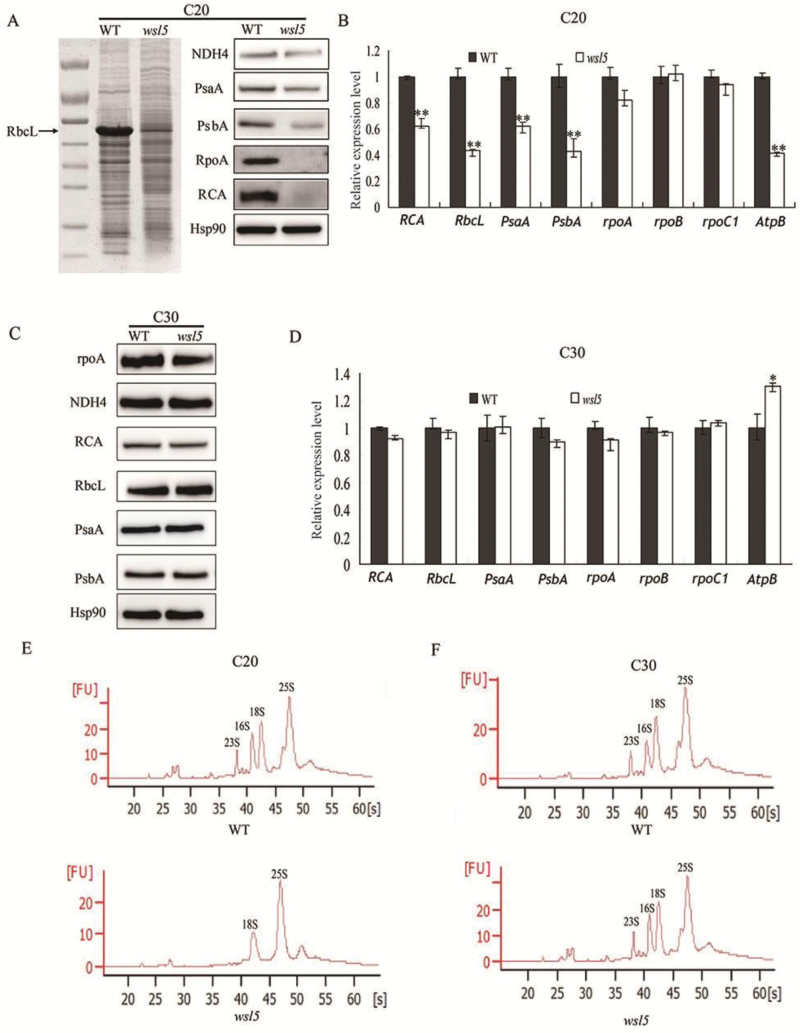
Analysis of accumulation of transcripts and proteins of representative genes associated with chloroplast biogenesis in wild-type (WT) and *wsl5* seedlings. (A, C) Western blot analysis of chloroplast proteins and RCA in WT and *wsl5* seedlings at the third-leaf stage at C20 (A) and C30 (C). Hsp90 was used as an internal control. (B, D) qRT-PCR analysis of relative expression levels of plastid-encoded genes in the WT and *wsl5* at the third-leaf stage under (B) C20 or (D) C30. Error bars represent the SD from three independent experiments. (E, F) rRNA analysis using an Agilent 2100 bioanalyzer. RNA was isolated from 10-day-old WT and *wsl5* seedlings grown at C30 and C20 (Student’s *t*-test, **P<0.01).

Since WSL5 was located in chloroplasts, we tested the accumulation of chloroplast proteins in *wsl5* and the wild type using western blot analysis under C20 and C30 conditons. Under C20 conditions, the protein levels of the large subunit of Rubisco (RbcL) and Rubisco activase (RCA) were much lower in *wsl5* (Fig. 7A). Other plastidic proteins including NADH dehydrogenase subunit 4, A1 of PSI, D1 of PSII, and the α-subunit of RNA polymerase were tested. The results showed that the levels of plastid-encoded proteins were significantly decreased in *wsl5* (Fig. 7A). Plastidic genes can be classified into three types. Class I genes are predominantly transcribed by PEP, class II genes are transcribed by both NEP and PEP, and class III genes are exclusively transcribed by NEP. qRT-PCR results suggested that the expression levels of class I genes *RbcL*, *psbA*, and *psaA* were strikingly reduced, whereas expression of the class III genes *rpoA* and *rpoC1*, and class II gene *AtpB*, was unchanged (Fig. 7B). When grown in C30 conditions, the transcripts and proteins of all genes in the mutant and the wild type showed very slight differences in expression pattern (Fig. 7C, D). These results indicated that *WSL5* was required for PEP activity under cold stress.

The chloroplast ribosome consists of a 50S large subunit and a 30S small subunit. Both subunits are comprised of rRNAs (23S, 16S, 5S, and 4.5S) and ribosomal proteins. We analyzed the composition and content of rRNAs using an Agilent 2100 bioanalyzer under C20 and C30 conditions. Both 23S and 16S rRNAs were decreased in *wsl5* seedlings under cold stress, but there was no difference relative to the wild type under C30 conditions (Fig. 7E, F). These results clearly indicated severe defects in plastidic ribosome biogenesis in the *wsl5* mutant seedlings grown under low temperature conditions.

### The *wsl5* mutant is defective in RNA editing and splicing of chloroplast group II introns

PPR proteins are required for RNA editing, splicing, stability, maturation, and translation (Tan *et al.*, 2014; Hammani *et al.*, 2016). Since WSL5 belongs to the P group, it was probably involved in transcript processing activities. First, we determined whether loss of WSL5 function affected editing at 21 identified RNA editing sites in chloroplast RNA (Corneille *et al.*, 2000). The results showed that the editing efficiencies of *rpl2* at C1 and *atpA* at C1148 were significantly decreased in the *wsl5* mutant compared with the wild type (Fig. S8 at Dryad) whereas the other nine genes and corresponding 19 editing sites were normally edited in the *wsl5* mutant. We then analyzed the editing efficiencies of *rpl2* at C1 and *atpA* at C1148 in complemented transgenic plants. As expected, the editing efficiencies of *rpl2* at C1 and *atpA* at C1148 were markedly improved in complemented plants (Fig. S8 at Dryad). These data supported the contention that the mutation in *WSL5* affected the editing efficiency of *rpl2* at C1 and *atpA* at C1148.

**Fig. 8.**
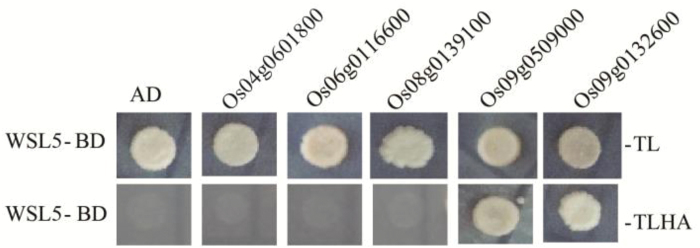
Yeast two-hybrid assay of WSL5 and MORF families. WSL5 was fused to the pGBKT7 vector (WSL5-BD). MORF protein was fused to the pGADT7 vector. LT, control medium (SD–Leu/–Trp); LTHA, selective medium (SD–Leu/–Trp/–His/–Ade). Empty pGBKT7 and pGAD-T7 vectors served as negative controls.

In *A. thaliana*, MORF proteins were implicated in RNA editing and provided a link between PPR proteins and proteins contributing to enzymatic activity (Takenaka *et al.*, 2012). Based on *A. thaliana* MORF protein families (Zehrmann *et al.*, 2015), we examined the potential interactions between rice MORF proteins and WSL5 by yeast two-hybrid analysis. The results showed that Os09g0509000 and Os09g0132600, both belonging to the *A. thaliana* MORF8 branch (Zhang *et al.* 2017), strongly interacted with WSL5 protein in yeast (Fig. 8). In contrast, Os04g0601800, Os06g0116600, and Os08g0139100 did not interact with WSL5 (Fig. 8). These results suggested that WSL5 may participate in RNA editing by interacting with OsMORF8s.

**Fig. 9.**
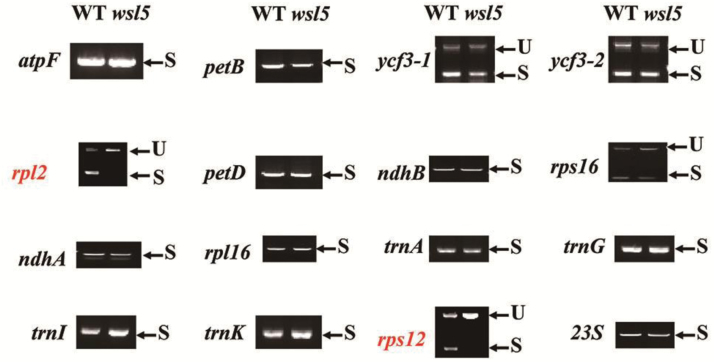
Splicing analysis of chloroplast transcripts in the wild type (WT) and *wsl5*. Gene transcripts are labeled on the left. Spliced (S) and unspliced (U) transcripts are shown on the right. RNA was extracted from WT and *wsl5* seedlings.

**Fig. 10.**
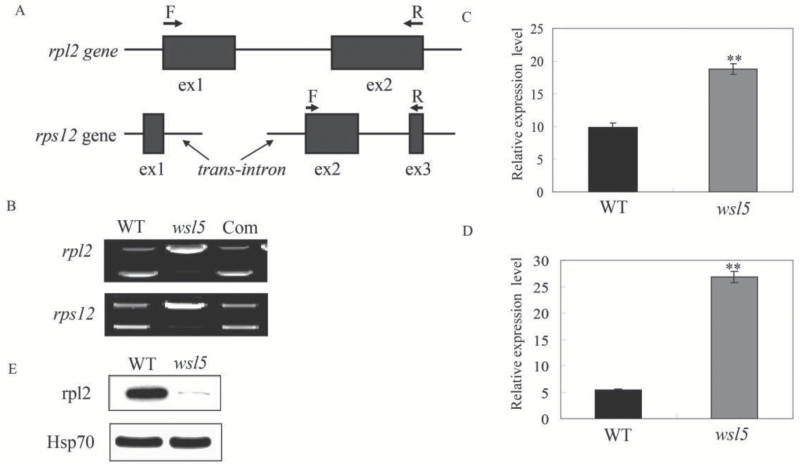
Splicing analysis of two chloroplast group II introns in the wild type (WT) and *wsl5*. (A) Sketch map of *rpl2* and *rps12* transcripts. (B) RT-PCR analysis of *rpl2* and *rps12* transcripts in the WT and *wsl5*. (C, D) qRT-PCR analysis of *rpl2* and *rps12* transcripts in WT and *wsl5* seedlings. (E) Western blot analysis of the rpl2 protein. Data are means ±SD of three repeats. Student’s *t*-test: **P<0.01.

We tested whether WSL5 is involved in RNA splicing of chloroplast genes. The rice chloroplast genome contains 18 introns (17 group II introns and one group I intron) (Kaminaka *et al.*, 1999). We amplified all chloroplast genes with at least one intron by RT-PCR using primers flanking the introns and compared the lengths of the amplified products between the wild type and the *wsl5* mutant. Chloroplast transcripts *rpl2* and *rps12-2* were spliced at very low efficiency in *wsl5* compared with the wild type (Figs 9, 10; Fig S9 at Dryad). To gain insight into the effects of the impaired splicing of *rpl2* and *rps12-2* on post-processing, we performed qRT-PCR and western blotting to examine the expression of *rpl2* and *rps12* in *wsl5* at the RNA and protein levels. The *rpl2* and *rps12* transcript abundances were high in the mutant compared with the wild type (Fig. 10C, D), although the rpl2 protein was at a low level in the mutant compared with the wild type (Fig. 10E). Thus, the low splicing efficiency of *rpl2* and *rps12-2* resulted in aberrant transcript accumulation and reduced levels of rpl2 protein in the *wsl5* mutant.

### Differentially expressed gene analysis in *wsl5* and the wild type under cold stress and normal conditions

**Fig. 11.**
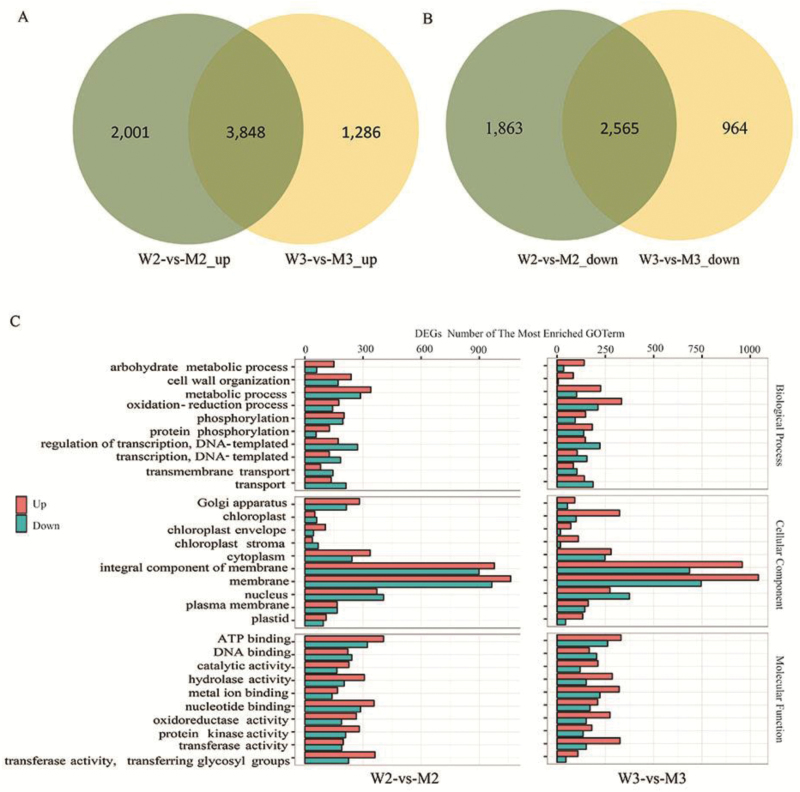
RNA-seq analysis of the wild type and *wsl5* grown under low temperature and normal conditions. (A) Up-regulated differentially expressed genes comparing W2 and M2 and W3 and M3. (B) Down-regulated differentially expressed genes comparing W2 and M2 and W3 and M3. (C) GO analysis of genes differentially expressed between W2 and M2 and W3 and M3. W3 and W2 represent wild-type plants grown at 30 °C and 20 °C, respectively. M3 and M2 represent *wsl5* plants grown at 30 °C and 20 °C, respectively.

To investigate why phenotypic variagation in the *wsl5* mutant depends on temperature, we carried out differential gene expression analysis in *wsl5* and wild-type seedlings grown in growth cabinets at C20 and C30 by RNA-seq. mRNA was purified from total RNA isolated from the third leaves using poly(T) oligo-attached magnetic beads; 6491 overlapping genes were up- or down-regulated between the two temperature treatments (Fig. 11A, B; Dataset S2 at Dryad). GO analysis indicated that the expression of genes involved in metabolic processes, oxidation–reduction processes, photosynthesis, light reaction, PSI and PSII, chloroplast thylakoid, ATP synthase, and carbon fixation were strongly reduced in the *wsl5* mutant at C20 (Fig. 11C). Functional plastidic ribosomes are crucial for the expression of the nuclear genes. However, the plastid could not assemble functional ribosomes because of the abnormal splicing of *rpl2* and *rps12* in the *wsl5* mutant. Retrograde signaling from chloroplasts to the nuclear genome plays an important role in plant chloroplast development. The altered expression of these nuclear genes may be due to blocked retrograde signaling.

## Discussion

### *WSL5* encodes a chloroplast-targeted PPR protein that is essential for chloroplast development in juvenile plants under cold stress

PPR genes constitute a large multigene family in higher plants. PPR proteins are essential for plant growth and development, and most of them are involved in RNA editing, splicing, and regulation of stability of various organellar transcripts (Barkan and Small, 2014). In contrast to PPRs in *A. thaliana*, little is known about the functions of PPRs in rice. Here, we present a molecular characterization of the PPR gene *WSL5* in rice. It has an RRM and 15 PPR motifs (Fig. S3 at Dryad). The WSL5 protein was predicted to contain a chloroplast transit peptide (cTP) in its N-terminal region, suggesting that the protein is one of the PPRs targeted to chloroplasts, and this was confirmed by subcellular localization experiments (Fig. 4D). The disruption of *WSL5* under natural conditions led to abnormal chloroplasts and caused a variegated phenotype that affected both the chlorophyll content and the chloroplast ultrastructure up to the four-leaf stage, whereas the *wsl5* mutant was albinic under cold stress (Fig. 2; Fig. S2 at Dryad). This finding suggests that the function of *WSL5* in rice is essential for early chloroplast development under cold stress. This conclusion is further supported by the results of expression analysis. *WSL5* was highly expressed in leaf sections L3 and L4 at the seedling stage. A high level of *WSL5* was noted under low temperatures. Sequence alignment of homologous proteins using *A. thaliana*, maize, and rice showed that the mutant site in *wsl5* is conserved within the RRM motif.

### WSL5 is involved in splicing of plastidic genes and in ribosome biosynthesis

A large group of nuclear-encoded PPR proteins involved in RNA editing, splicing, stability, maturation, and translation is required for chloroplast development (Tan *et al.*, 2014; Wang *et al.*, 2017). To date, six PPR proteins have been reported to be involved in RNA splicing of group II introns in chloroplasts. Among them, the maize PPR4 protein acts as an *rps12* trans-splicing factor (Schmitz-Linneweber *et al.*, 2006). *Arabidopsis thaliana* PPR protein OTP51 functions as a plastid *ycf3-2* intron *cis*-splicing factor and OTP70 has been implicated in splicing of plastid transcript *rpoC1* (de Longevialle *et al.*, 2008; Chateigner-Boutin *et al.*, 2011). In this study, the *wsl5* mutant caused defects in the splicing of *rpl2* and *rps12* in rice (Figs 9, 10), implying that WSL5 probably controls chloroplast RNA intron splicing during early leaf development. The majority of those splicing factors act on distinct, but overlapping, intron subsets, and each intron has been shown to require multiple proteins (de Longevialle *et al.*, 2010; Khrouchtchova *et al.*, 2012). In rice, WSL can splice *rpl2*, while WSL4 can splice *rpl2* and *rps12* (Tan *et al.*, 2014; Wang *et al.*, 2017). WSL5 could not interact with WSL and WSL4 in the yeast system (data not shown). These three proteins are all involved in the splicing of *rpl2*. There is no functional redundancy among them. There may be an unknown regulatory mechanism to control the function of these three proteins. Further studies on searching for interacting partners of WSL5 will help to uncover the regulatory mechanism of the splicing of chloroplast genes.

Defective *rps12* and *rpl2* splicing could account for the white-stripe leaf phenotype and plastid ribosome deficiency in the *wsl5* mutant (Figs 9, 10). We found that 23S and 16S rRNAs were decreased in the *wsl5* mutant under cold stress (Fig. 7E, F). The lack of mature *rps12* and *rpl2* mRNA in the *wsl5* mutant may severely affect ribosome functions in plastids. The absence of RPL2 and RPS12 protein resulted in the inability to make functional ribosomes. Thus, the ribosome assembly defect in *wsl5* may also contribute to the *wsl5* phenotype.

### Possible mechanism of *WSL5* regulating chloroplast development under cold stress and normal conditions

To study the molecular mechanism of *WSL5* in regulating chloroplast development under different temperature conditions, we compared gene expression patterns in the *wsl5* mutant and wild type by RNA-seq analysis. Our findings showed that under cold stress, *WSL5* influences the expression of genes involved in carbohydrate metabolism, oxidation–reduction processes, photosynthesis, biosynthesis of secondary metabolites, chlorophyll biosynthesis process, and chloroplast development (Fig. 11; Dataset S2 at Dryad). Plastid thioredoxins are important for maintaining plastid oxidation–reduction balance (Bohrer *et al.*, 2012). Many genes involved in regulating plastid oxidation–reduction balance were changed under C20 and C30 conditons, such as *OsTRXm* and *OsTRXz* (Fig. S10 at Dryad). *OsTRXm* is involved in regulation of activity of a target peroxiredoxin (Prx) through reduction of cysteine disulfide bridges (Chi *et al.*, 2008). OsTRXz interacts with TSV to protect chloroplast development under cold stress (Sun *et al.*, 2017). The large and small subunits of ribonucleotide reductase (RNR), V3 and St1, regulate the rate of deoxyribonucleotide production for DNA synthesis and repair (Yoo *et al.*, 2009). *V3* and *St1* are repressed at constant 20 °C in *wsl5*, indicating that mutation in *WSL5* leads to defects in DNA synthesis and repair in juvenile plants at low temperatures (Fig. S10 at Dryad). The expression of fatty acid metabolism genes *OsFAH1*, *OsFAH2*, and *OsFAD7*, and plastid starch metabolism genes *AGPS2b* and *PHO1*, was dramatically changed in *wsl5* compared with the wild type at low temperatures (Fig. S10 at Dryad). Chloroplast development depends on co-operation between nuclear and chloroplast genes. The altered expression of these nuclear genes in the *wsl5* mutant may be due to the absence of RPL2 and RPS12 proteins and thereby impede the transfer of retrograde signaling from plastids to the nucleus.

In conclusion, *WSL5* influences the expression of plastid genes and biogenesis of plastid ribosomes. It is essential for chloroplast development in rice seedlings under cold stress by maintaining the retrograde signaling from plastids to the nucleus. Identification of this new PPR protein will help to elucidate the molecular mechanisms of plastid development and ribosome biogenesis, and shed light on understanding chloroplast development in juvenile plants under cold stress.

## Data deposition

The following data are available at Dryad Data Repository: http://dx.doi.org/10.5061/dryad.59185.

**Dataset S1.** Genes differentially expressed in the wild type and *wsl5*.

**Dataset S2.** Genes differentially expressed in the wild type and *wsl5* under different temperature conditions.

Fig. S1. Comparison of pigment contents from the second (L2), third (L3), fourth (L4), and fifth (L5) leaves of five-leaf stage plants between the wild type and the *wsl5* mutant.

Fig. S2. Transmission electron microscopy images of cells from the wild type and the *wsl5* mutant grown under different temperature conditions.

Fig. S3. Alignment of *WSL5* orthologs in maize and Arabidopsis.

Fig. S4. *WSL5* was expressed in all tissues, especially during leaf development, according to Rice eFP Browser.

Fig. S5. GO analysis of genes differentially expressed between the wild type and *wsl5.*

Fig. S6. Pathway analysis of genes differentially expressed between the wild type and *wsl5.*

Fig. S7. Expression levels of chlorophyll synthesis genes in the wild type and *wsl5*.

Fig. S8. Editing efficiencies of *rpl2* and *atpA* in the wild type and the *wsl5* mutant.

Fig. S9. qRT-PCR analysis of *rpl2* and *rps12* transcripts in the wild type and the *wsl5* mutant.

Fig. S10. qRT-PCR analysis of genes differently expressed in RNA-seq.

Table S1. Comparison of agronomic traits between the wild type and *wsl5* under field conditions.

Table S2. Primers used in this study.
