# Corrigendum: WSL5, a pentatricopeptide repeat protein, is essential for chloroplast biogenesis in rice under cold stress

**DOI:** 10.1093/jxb/ery259

**Published:** 2018-07-14

**Authors:** Xi Liu, Jie Lan, Yunshuai Huang, Penghui Cao, Chunlei Zhou, Yaken Ren, Niqing He, Shijia Liu, Yunlu Tian, Thanhliem Nguyen, Ling Jiang, Jianmin Wan

**Affiliations:** 1 State Key Laboratory for Crop Genetics and Germplasm Enhancement, Jiangsu Plant Gene Engineering Research Center, Nanjing Agricultural University, Nanjing, China; 2 National Key Facility for Crop Gene Resources and Genetic Improvement, Institute of Crop Science, Chinese Academy of Agricultural Sciences, Beijing, China

*Journal of Experimental Botany,* Advance Access publication: June 8, 2018. doi:10.1093/jxb/ery214

The original online version of this article contained an outdated figure (Figure 7). This figure has now been corrected.

The authors would like to apologise for this error.

